# Interactions of Bacteria With Monolithic Lateral Silicon Nanospikes Inside a Microfluidic Channel

**DOI:** 10.3389/fchem.2019.00483

**Published:** 2019-07-12

**Authors:** Lei Li, Feng Tian, Hao Chang, Jie Zhang, Cheng Wang, Wei Rao, Huan Hu

**Affiliations:** ^1^CAS Key Lab of Cryogenics, Technical Institute of Physics and Chemistry, Chinese Academy of Sciences, Beijing, China; ^2^ZJU-UIUC Institute (ZJUI), Zhejiang University, Haining, China; ^3^College of Information Science and Electronic Engineering, Zhejiang University, Hangzhou, China; ^4^Department of Mechanical and Aerospace Engineering, Missouri University of Science and Technology, Rolla, MO, United States; ^5^School of Future Technology, University of Chinese Academy of Sciences, Beijing, China

**Keywords:** lateral silicon nanospikes, nanofabrication, metal-assisted chemical etching, anti-bacterial, lab on a chip

## Abstract

This paper presents a new strategy of integrating lateral silicon nanospikes using metal-assisted chemical etching (MacEtch) on the sidewall of micropillars for on-chip bacterial study. Silicon nanospikes have been reported to be able to kill bacteria without using chemicals and offer a new route to kill bacteria and can prevent the overuse of antibiotics to reduce bacteria. We demonstrated a new methodology to fabricate a chip with integrated silicon nanospikes onto the sidewalls of micropillars inside the microfluidic channel and attested its interactions with the representative gram-negative bacteria *Escherichia coli*. The results of colony-forming unit (CFU) calculation showed that 80% bacteria lost their viability after passing through the chip. Moreover, the results of adenosine triphosphate (ATP) measurement indicated that the chip with lateral silicon nanospikes could extract more than two times ATP contents compared with the chip without lateral silicon nanospikes, showing potential for using the chip with lateral silicon nanospikes as a bacterial lysing module.

## Introduction

Lab-on-a-chip (LOC) technology, which features microchannels and microstructures with dimensions ranging from a micron to a few 100 microns, has offered tremendous benefits for the research of mammalian cells since early 1990s. In recent years, increasing numbers and varieties of LOC devices have been used for bacteria study, such as capture (Guo et al., 2012), separation (Beech et al., 2018), and detection (Jalali et al., 2018). However, since the size of bacteria is usually about one micron and the widely used ultra-violet (UV) light lithography for LOC fabrication cannot produce structures below one micron due to diffraction limit, the performance of LOC systems for bacterial research have not reached the same level with mammalian cells.

Introducing nanostructures with the similar size as bacterial cells into LOC systems can interact with individual bacteria for more efficient manipulation, separation and lysing, and eventually achieve single bacteria analysis. The fabrication and integration of nanostructures on a chip generally require top-down nanofabrication technology, such as electron beam lithography and focused ion beam. However, these top-down nanofabrication technologies generally require expensive equipment and are not scalable. Therefore, they are not suitable for fabricating microfluidic chip with nanostructures in a cost-effective and affordable approach.

Along another route, nanomaterials such as nanoparticles (Gao et al., 2014), nanowires (Jeong et al., 2013; Liu et al., 2018), carbon nanotubes (Akasaka and Watari, 2009), and nano diamonds (Wehling et al., 2014; Ong et al., 2018) have been studied about their interactions with bacteria (Yang et al., 2008). One application is the construction of antibacterial surfaces. Recently, scientists have discovered a new method of killing bacteria using nanospikes on wings of insects such as cicadas and dragonflies (Ivanova et al., 2012). Ivanova et al. suggested The bacterial killing mechanism or bactericidal mechanism was purely due to the morphology and the interaction between the spikes and the membrane of bacteria. Adsorption of bacterial membrane on the nanospike surfaces leaded to drastic increase of contact area, accompanied by stretching of the bacterial membrane, likely to cause irreversible membrane rupture and death of bacteria (Pogodin et al., 2013). Similar nanostructures but made of other materials such as titanium (Bhadra et al., 2015), titanium oxide (Diu et al., 2014), diamond (Fisher et al., 2016), copper oxide (Nishino et al., 2017), gold (Wu et al., 2016), and zinc oxide (Sengstock et al., 2014) also show anti-bacteria properties, which indicates that the bactericidal mechanism is highly related to the structure morphologies but less related to the surface chemistry. In addition, materials with only one dimension in nanometers are also reported to be capable of killing bacteria. Tu et al. (2013) reported killing bacteria using materials such as graphene and graphene oxide. Molecular dynamic simulation also revealed that it was energetically favorable for the graphene sheets to enter the hydrophobic interfaces of two contacting proteins, leading to disrupted metabolisms of cells (Luan et al., 2015). Previously, we also demonstrated antibacterial surfaces by vertical silicon nanospikes on bare silicon samples (Hu et al., 2017).

LOC devices with some similar nanostructures have been reported but mostly are for the study of mammalian cells. For example, Di Carlo et al. (2003) used silicon nanoscale barbs formed during deep reactive ion etching of silicon to lyse human leukemia cells. Kim et al. (2012) used ZnO nanowires grown on the sidewall of PDMS micropillars to disrupt HaCaT, HeLa and Jurkat cells. So et al. (2014) used ZnO nanowires-decorated multifunctional membrane for a hand-held cell lysis devices. Yun et al. (2010) used silicon nano-blade arrays produced by wet etching of (110) silicon for EL4 mouse T-lymphoma cells disruption. However, it is worth noting that these studies are all based on mammalian cells and to the best of our knowledge, this kind of devices has not been used for bacterial study. It is well-known that bacterial cells and mammalian cells have differences in both of their sizes and the structures of membranes. Therefore, it is both interesting and fundamentally important to investigate how bacterial cells will response and react to these sharp nanostructures on a LOC platform.

Here, we present a hybrid strategy to integrate silicon nanospikes prepared by metal-assisted chemical etching (MacEtch) on the sidewalls of silicon micropillars fabricated by microfabrication technology, creating a unique environment for the study of interaction of bacteria with sharp nanostructures on a chip. The method of producing the nanospikes, MacEtch, is a low-cost and scalable nanofabrication method (Li, 2012). The results of colony-forming unit (CFU) calculation of bacterial sample flowing through devices containing lateral silicon nanospikes showed 80% reduction compared to the original sample. ATP measurements of bacterial sample also showed more than two times increase of ATP concentration compared with using chip without nanospikes, which represents that More contents were released from bacteria. Scanning electron microscope images of devices after test also revealed both captured bacteria and lysed bacteria. Our work presents a new methodology to synergistically combine bottom-up nanofabrication technology for nanostructures and top-down microfabrication technology for microstructures to produce a cost-effective LOC with integrated nanostructures for bacterial killing and assess the possibility of using nanospike-based approaches for bacteria lysing.

## Experiments

### Fabrication Process

**Figure 1:**
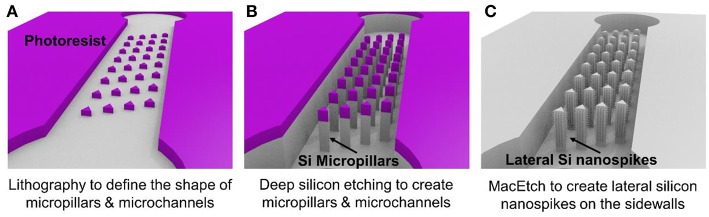
Major steps of the fabrication process for the chip with silicon lateral nanospikes. **(A)** UV lithography to define the shapes of micropillars and the microchannels; **(B)** deep reactive ion etching of silicon and silicon oxide; **(C)** MacEtch to produce lateral silicon nanospikes on the sidewalls and remove oxide using hydrofluoric acids.

Figure 1 illustrates the three major steps of the fabrication process. Using conventional lithography and deep reactive ion etching, silicon microchannels and silicon micropillars were fabricated on a single crystal silicon wafer with (100) crystal direction as shown in Figures 1A,B. Then MacEtch was applied to fabricate silicon nanospikes on sidewalls of silicon micropillars. The top part of the silicon substrate is protected by the 2 μm thick silicon oxide. After silicon nanospikes were created on the sidewalls, we used hydrofluoric acid to remove the top silicon oxide. Finally, a flat piece of PDMS cover with pre-punched inlets and outlets was bonded on top of the silicon substrate to render the chip.

For the MacEtch process, we used a mixture solution of 20 mM silver nitrate and 5 M hydrofluoric acid at room temperature. Prior to MacEtch, the silicon sample with micropillars was placed in a furnace to grow 500 nm thick thermal oxide and etched with hydrofluoric acid to remove the scallops or the roughness on the sidewalls of the micropillars. This sidewall smoothing step is critical for producing silicon nanospikes (Gao et al., 2014). Then the sample was dipped into the MacEtch etchants to produce lateral silicon nanospikes on the sidewalls of the micropillars (shown as Figure 1C). The tip radius of nanospikes fabricated by MacEtch were reported to be distributed from 20 to 300 nm with an average of 100 nm (Hochbaum et al., 2008) and were sharp enough to disrupt the cell membranes as reported.

Detailed fabrication procedure was provided in Figure S1. We also fabricated a microfluidic chip with only silicon micropillars (shown as Figure 1B) and bonded with a PDMS cover to be acted as the control device.

### Numerical Simulation and Design Optimization

**Figure 2:**
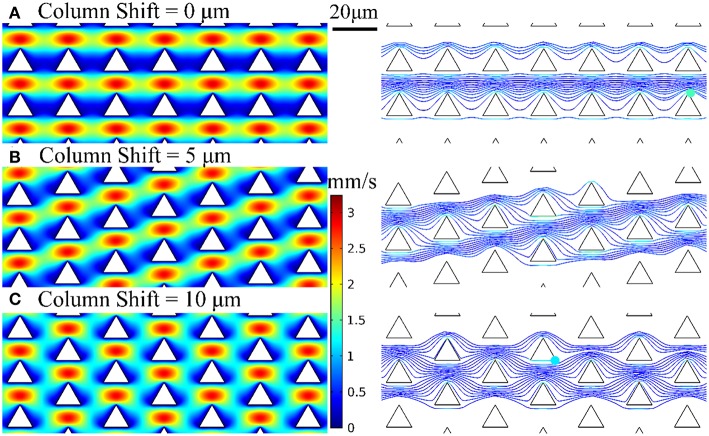
Velocity profiles and bacteria trajectories for three configurations of triangular-shaped micropillars of column shift of **(A)** 0 μm, **(B)** 5 μm, and **(C)** 10 μm.

We employed the design of deterministic lateral displacement (DLD) device (Huang et al., 2004) that uses an array of micropillars to manipulate the laminar flow streams inside the microfluidic channels. This strategy facilitates the flow streams around the edges of micropillars to increase the chances of bacteria to contact the micropillars and have been used for separating mammalian cells (Inglis et al., 2011), bacteria (Inglis et al., 2010), DNA (Huang et al., 2004), and exosomes (Kim et al., 2017). The micropillar geometry has a significant impact on the flow stream and affect collision probability with the micropillars and thus the chance of bacteria being captured by the nanospikes. Therefore, we used numerical simulations to optimize the design of silicon micropillar shapes for increased collision probability and improved capturing efficiency. The Navier-Stokes equation and Newton's second law were used to model the flow field and bacteria trajectory in the microchannel, respectively. Briefly, the flow field is first solved by a stationary solver, and then the particle tracing for fluid flow is solved by a time-dependent solver. The effects of three different configuration of triangular-shaped micropillars on bacteria capturing efficiency and collision probability are investigated. The numerical results of velocity profiles and bacteria trajectories are shown in Figure 2. The column shifts of micropillars configuration in Figures 2A–C are 0, 5, and 10 μm, respectively. The bacteria capturing efficiency is correlated to the percentage of bacteria that enter certain distance range (2 μm) from the micropillars, by assuming finite size of *Escherichia coli* bacteria. Based on this criterion, the triangle configuration of column shift by 5 μm provides the maximum collision efficiency. Therefore, we chose the design of triangular micropillars with 5 μm column shift.

**Figure 3:**
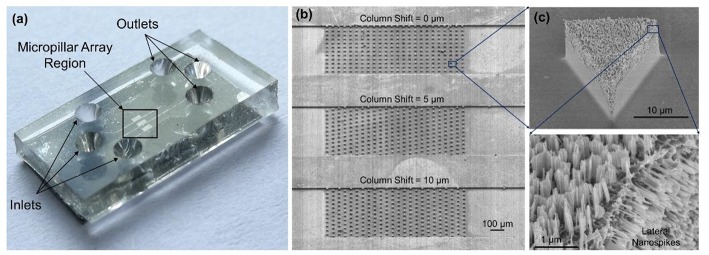
**(a)** Photo of the completed device with PDMS cover on top of the silicon chip. **(b)** SEM images of three separate microchannels each containing an array of micropillars with different pillar arrangements. **(c)** SEM image of a triangular-shaped micropillars with lateral and top silicon spikes.

Figure 3a shows the photo of a completed device. Each device has three separate microchannels with micropillar regions in the middle of channels serving as the interaction zone. Figure 3b shows the SEM images of the fabricated microchannels and micropillars. Figure 3c shows a triangular-shaped micropillar with lateral silicon spikes on the sidewalls. The lateral spikes have a tip radius in the range of 10–200 nm, which could produce shear force to disrupt the bacterial cell walls under shear flows.

### Bacteria Test Procedures

**Figure 4:**
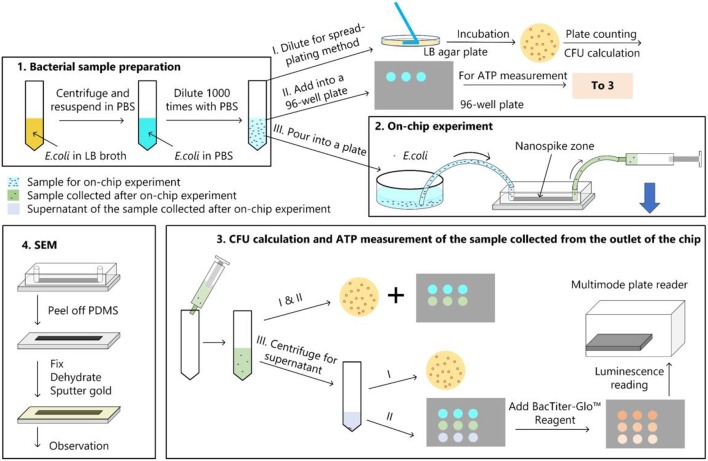
Bacterial experimental protocol. (1) Bacterial sample preparation; (2) On-chip experiment; (3) CFU calculation and ATP measurement for the samples collected from the chip; (4) main procedures of the sample preparation for the SEM observation.

The bacterial killing ability of the chip was quantified by comparing the numbers of colonies of the samples with and without chip treatment with spread-plating methods. To determine whether the bacterial cells were lysed and released their contents, we measured the amount of ATP from the supernatant of the solution passed through the chip. In addition, We used a scanning electron microscope to verify the bacteria capture and lysing qualitatively. The experimental procedure was represented in Figure 4.

#### Bacterial Sample Preparation

Luria-Bertani (LB) broth and LB agar were prepared with tryptone (Oxoid Ltd., UK), yeast extract (Oxoid Ltd., UK), NaCl (Sinopharm Chemical Reagent Co Ltd., China), agar (Becton, Dickinson and Company, USA), and distilled water. *E. coli* DH5α cells incubated in LB broth were harvested at exponential phase and the viable cells were counted by quantifying the CFUs with spread-plating method. The initial bacterial concentration was about 10^9^ CFU/mL.

The cells were centrifuged at 4,000 rpm for 5 min and rinsed with PBS buffer solution (Thermo Fisher Scientific, pH 7.4) for three times. Then the bacterial sample was diluted 1,000× in PBS for the subsequent on-chip experiments. Bacterial cell numbers of the diluted sample were determined by plate counting of CFUs on LB agar plates and ATP measurement.

#### On-Chip Experiment

As shown in Figure 4, the diluted bacterial sample was poured into a plate and silicone tubes were inserted into the inlet and outlet of chip. Then the inlet tube was inserted into the liquid and the outlet tube was connected to a syringe. The syringe was withdrawn and held for 10 min to get the solution passing through the chip. Approximate 500 μL of liquid was collected from the outlet, which indicated a flow rate of about 50 μL/min. Bacterial cell number of the sample after on-chip experiment was determined by spread-plating method and ATP measurement.

#### ATP Measurement

The amount of ATP was measured by using the BacTiter-Glo^TM^ Microbial Cell Viability Assay (Promega, USA). The luminescent signal is proportional to the amount of ATP present. In addition to measure the ATP amount of the sample after chip treatment, we also centrifuged the sample at 4,000 rpm for 10 min to get the ATP amount of the supernatant. Luminescence for the supernatant (L_SUP_) and the sample (L_SAM_) were recorded on a multimode plate reader (EnSpire, PerkinElmer, USA).

#### SEM Observation

After the on-chip experiment, we peeled off the PDMS layer and soaked the silicon structure into 4% glutaraldehyde (Beijing Leagene Biotech Co Ltd, China), dehydrated in 50, 70, 80, 90, 95, and 100% ethanol. Then the silicon structure was dried and covered with a thin gold film by using an Ion Sputter Coater (MC1000, Hitachi, Japan) before imaging by the environmental scanning electron microscope (QUANTA FEG 250, FEI, Hillsboro, OR, USA).

#### Statistical Analysis

The one-way analysis of variance (ANOVA) with Tukey HSD test was used to determine if there were statistically significant differences between the means of the groups. Data were expressed as the mean ± SD from three independent on-chip experiments.

## Results and Discussions

**Figure 5:**
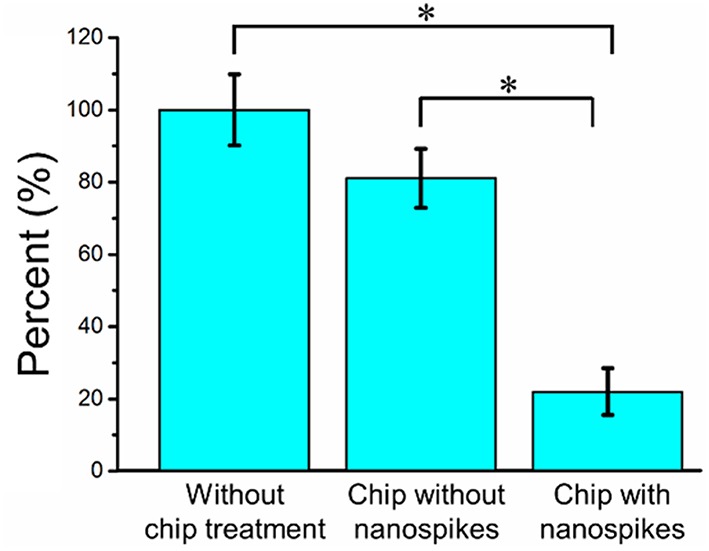
Bacterial killing performance as indicated by the decreases in CFU compared with the chip without nanospike (control). The concentration of the input bacterial sample was ~2.38 × 10^6^ CFU/mL. *Significant difference between groups (*p* < 0.01).

We first compared the CFU of the bacterial solution collected from the outlet of the chip with that of the sample without chip treatment (shown as numbers of percentages in Figure 5 and the CFU of the sample without chip treatment was used as 100%). The chip without silicon nanospikes on the sidewalls of micropillars were tested as the control group. For the device without silicon nanospikes but only silicon micropillars, the CFU of output sample decreased about 19%, while for the chip with silicon nanospikes, the CFU significantly decreased about 80%. There was no statistically significant difference (*p* > 0.05) between the group without chip treatment and the group with only micropillars inside the chip. In contrast, there were statistically significant decreases in viable cell number (*p* < 0.01) after passing through the chip with lateral nanospikes. The existing of nanospikes remarkably increased the killing efficiency of the bacterial cells. The results also indicated that most bacteria interacted with the lateral silicon nanospikes when they flew through the chip and lost their viability. When the concentration of the bacterial solution increased to about five times, CFU decreased 63.5% (shown as Figure S2), indicating that the chip has its limit for bacteria processing. Since the chip has fixed amount of lateral silicon nanospikes and can only process a certain number of bacteria, with bacteria concentration increasing, the average collision efficiency will decrease and thus less bacteria get killed.

**Figure 6:**
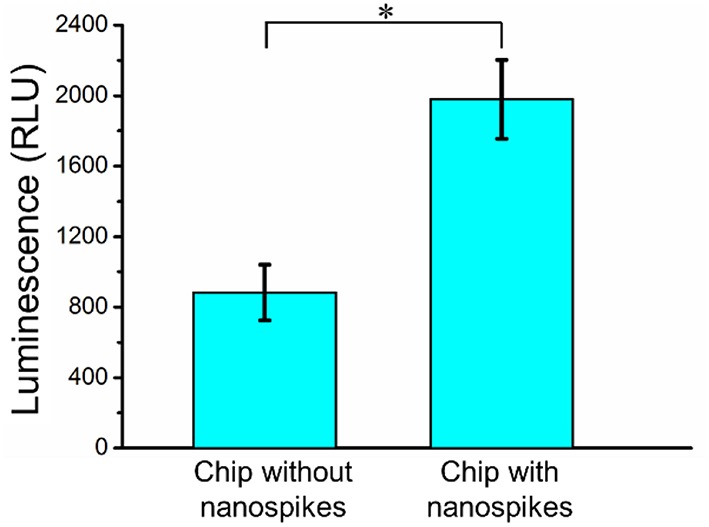
ATP measurement results of the sample passed through the chip with and without nanospikes. *Significant difference between groups (*p* < 0.01).

**Figure 7:**
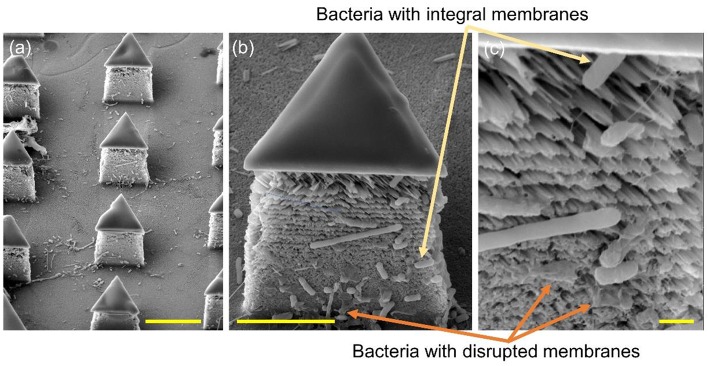
SEM images of the silicon micropillars with lateral silicon nanospikes. **(a)** an array; **(b)** zoomed-in view of a single triangular micropillar; **(c)** zoomed-in view of bacteria observed sticking on the lateral silicon nanospikes, some of them still had integral cell walls while some of them had been disrupted. Scale bars are 10, 5, and 1 μm for **(a–c)**, respectively.

To further determine the lysing capability of the chip, we centrifuged the output samples for their supernatant and test the content of extracellular ATP. We supposed that if the bacterial cells were disrupted by the nanospikes when they passed through the microchannel, their ATP would release into the solution. The results of the luminescence of the supernatants were shown in Figure 6, which suggested that the chip with lateral silicon nanospikes lysed more than two times more of the bacterial cells than the chip with only micropillars and there was a statistically significant difference between the two groups (*p* < 0.01). In addition, we also investigated that some *E*. *coli* bacteria cells were captured on the sidewalls and some of them appeared to be lysed with the disrupted membrane from the SEM images of an array of triangular micropillars with silicon nanospikes (shown in Figure 7).

In this work, MacEtch was used to fabricate silicon nanospikes on the sidewalls of micropillars in the microchannel. Compared with other nanofabrication methods, MacEtch shows significant advantages. First, MacEtch offers the benefits of lower cost and lower power consumption. It only requires regular chemicals and can be operated in room temperature without using sophisticated vacuum equipment (Linklater et al., 2017; Du et al., 2018; Hazell et al., 2018) or consuming a big amount of power for high-temperature, which leads to significant power saving and green manufacturing. Second, MacEtch offers the unique capability of fabricating monolithic nanostructures on the sidewalls of silicon microstructures, which is very challenging for conventional semiconductor manufacturing technologies. Fabricating nanostructures on the sidewall can enable improved functionalities, such as the better liquid repelling capability for the self-cleaning surface. Conventional semiconductor manufacturing, such as reactive ion etching, can only produce micro/nanostructures on the front surface of the sample and has limited access to the sidewall. Other bottom-up methods, such as growing nanostructures and attaching nanomaterials on the surfaces, tend to have adhesion issues because the nanomaterials are attached to the surface via relatively weak Van der Waals force (Yun et al., 2010).

This device can kill about 80% of the bacterial cells by using mechanical structure without using chemicals. Several studies suggest that the cell membrane ruptured by nanospikes is purely due to physical disruption, as Pogodin et al. (2013) and Li (2016) proposed in their papers. Adsorption of bacterial membrane on the nanospike surfaces led to a drastic increase of contact area, accompanied by stretching of the bacterial membrane, likely to cause irreversible membrane rupture and death of bacteria. Moreover, the chip can also potentially function as a bacterial lysing module and can be integrated with other modules such as separation and analysis to form a LOC system performing a complete set of operation for bacteria analysis. This is particularly useful at places where bacterial lysing agents are not available and other types of lysing technique such as acoustic or electrical are difficult to implement. However, the lysing efficiency with the current chip was only around 16.67% (detailed calculation process was shown in the Supplemental Material), meaning that 16.67% of the bacterial cells actually had their membranes disrupted and released their contents despite the fact that 80% of the cells lost their viability after passing through the chip. This might be attributed to the structure of cell wall of the gram-negative bacterial species, which consists of three layers–the outer and inner cytoplasmic membranes, and a peptidoglycan layer in between. Therefore, it is much more challenging to disrupt their cell walls compared to mammalian cells which only have the plasma membrane consisting of mainly bi-lipid layers. The membrane of bacterial cells may be partially damaged, which induced the loss of viability and the capability for growing into colonies, but insufficiently to release their contents.

To improve the bacterial lysing efficiency, we could increase the shear force acted upon the bacterial cells by increasing the flow rate of sample and further reduce the tip radius of nanospikes by optimizing thermal oxidation and hydrofluoric acid etching (Resnik et al., 2003). Moreover, we could increase the width and length of the microchannels to arrange more micropillars to increase the collision probability when the bacterial cells flowing through the chip. As shown in Figure 7, some dead bacterial cells covered the nanospikes and prevented subsequent puncture. Therefore, increasing the height of micropillars for larger areas of nanospikes and implementing backwashing to remove the some of the dead bacteria cells might be helpful for a higher processing capability. Further works are on-going to optimize the chip design and process conditions for the improvement of the lysing efficiency as well as the throughput.

## Conclusions

We presented a new methodology to fabricate a chip with monolithic lateral silicon nanospikes on sidewalls of silicon micropillars inside a microfluidic channel. Our chip provides a unique tool to study the interactions of bacteria with sharp nanospikes. Our measurement results showed that this chip killed 80% of *E. coli* based on mechanical forces during the interaction of the bacteria and the nanospikes. Moreover, the results also suggested that this chip could further be served as a mechanical lysing module and integrated with other functional modules such as separation and analysis to form a whole-functioning bacterial test chip.

## Data Availability

All datasets generated for this study are included in the manuscript/Supplementary Files.

## Author Contributions

HH initiated the concept and designed and fabricated the chip. LL designed the bacteria test protocol and performed the test. FT contributed to the device fabrication. HC and WR contributed in bacteria growth and test. JZ and CW performed the flow simulation of the chip. LL, CW, and HH drafted the manuscript together.

### Conflict of Interest Statement

The authors declare that the research was conducted in the absence of any commercial or financial relationships that could be construed as a potential conflict of interest.
