# Supplementary material for: Interactions of Bacteria With Monolithic Lateral Silicon Nanospikes Inside a Microfluidic Channel
Source: Front Chem. 2019 Jul 12;7:483. doi: 10.3389/fchem.2019.00483 (PMC6640657; doi:10.3389/fchem.2019.00483)
Supplement: Supplementary file 1 [file Data_Sheet_1.PDF]

Supplemental Materials

I. Fabrication Process

Figure S1 shows the fabrication process of our chip with lateral silicon spikes in detail. The process starts with a (100) single-crystal silicon wafer with 2um thick silicon oxide prepared by wet thermal oxidation of silicon (step 1). Then UV-lithography was used to define the shape of microfluidic channels as well as micropillars (step 2). Deep silicon etching was then used to etch exposed region of 2um thick silicon oxide and 20um thick silicon to form microchannels and micropillars (step 3). After the silicon etching step, we removed the photoresist using acetone and oxygen plasma (step 4). At this step, silicon microfluidic channels and silicon micropillars are completed (step 4).

The next step is to fabricate silicon nanospikes using metal-assisted chemical etching. To ensure good quality of silicon nanospikes, a 200nm thick silicon oxidation step followed by hydrofluoric acid etching were performed to smooth the sidewall of silicon micropillars (step 5&6). Then the silicon substrate was treated with MacEtch solution for 8 minutes to create silicon nanospikes on the sidewalls of micropillars. The top surface of the silicon chip was protected by silicon oxide. Finally, a PDMS cover layer with pre-punched inlets and outlets were bonded to the silicon substrate to render the chip.

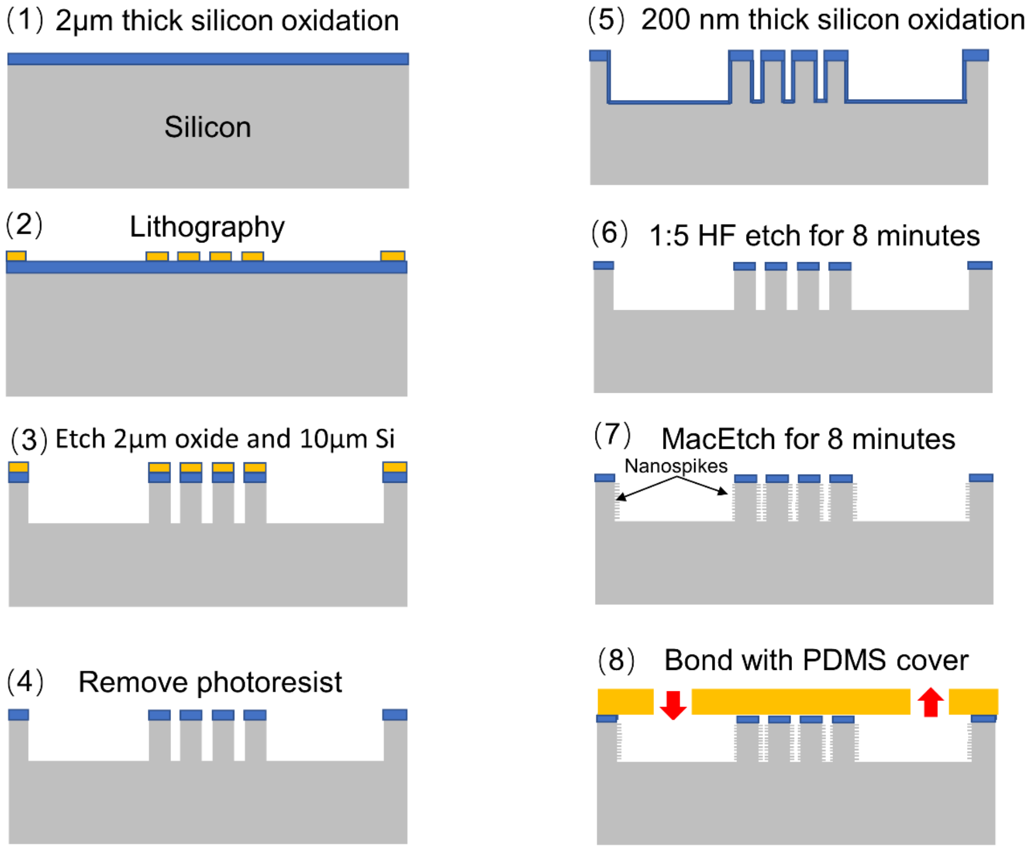

Figure S1. Detailed processing steps for fabricating the chip.

II. CFU calculation

We increased the concentration of input bacterial solution to about 5 times (about 1.2×10^6 CFU/mL from the CFU calculation results) and test this sample on the chip with nanospikes as well as the chip without nanospikes. CFU of the output sample of the chip without nanospikes was about 84.62% and that of the chip with nanospikes was about 36.54% (shown as figure S2).

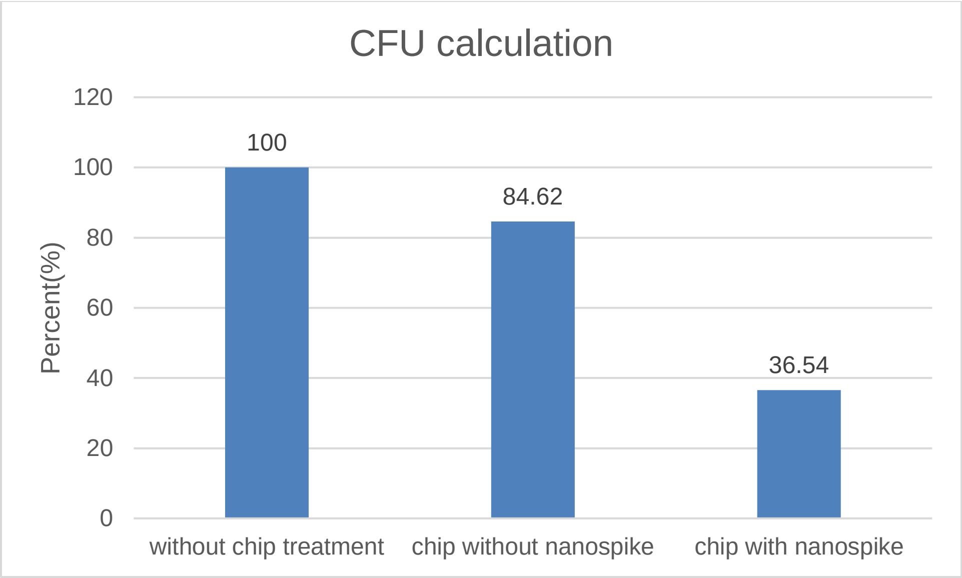

Figure S2. Bacterial killing performance as indicated by the decreases in CFU. The concentration of the input bacterial sample was about 1.2×10^6 CFU/mL.

III. The calculation of the lysing efficiency from the ATP measurement

The luminescence of the supernatant (L_{SUP}) of the sample passed through the chip with nanospikes was 1980 ± 223, as shown in figure 6. The luminescence of the input sample (L_{SAM}) was 11880 ± 197 (data not shown in paper). The lysing efficiency was calculated as the (L_{SUP}/ L_{SAM}) × 100%, which was about 16.67%.
