# Supplementary material for: Interactions of Bacteria With Monolithic Lateral Silicon Nanospikes Inside a Microfluidic Channel
Source: Front Chem. 2019 Jul 12;7:483. doi: 10.3389/fchem.2019.00483 (PMC6640657; doi:10.3389/fchem.2019.00483)
Supplement: Supplementary file 2 [file Data_Sheet_2.PDF]

Supplemental Materials

Q1: What is the pressure applied by the syringe? Can you change the killing efficiency by changing the flow rate?

When applying pressure to the channel by syringe, we withdrew the syringe and held for 10 min for the bacterial solution passing through the chip. The pressure applied by the syringe can be calculated by the following formula:

$$Q = \frac{4\Delta pbc^3}{\mu L}\left[\frac{1}{3} - \frac{64}{\pi^5}\frac{c}{b}\tanh\left(\frac{\pi b}{2c}\right)\right] \qquad (1)$$

where b *is the half channel width,* c *is the half channel height,* L *is the channel length,* μ *is the fluid viscosity,* v_{avg} *is the average velocity, and* Δp *is the pressure required. In our experiment,* b = *120 µm,* c = *5 µm,* L = *750 µm, and* Q *= 50 µL/min. And the dynamic viscosity* μ *is estimated to be similar as water 0.001 N·S/m*^{2}*.*

The estimated pressure is about 300 kPa. With micropillars in a small portion of the channel, the pressure also goes up a bit but stays in the same level.

(1) F. M. White, Viscous Fluid Flow, ch. 3. New York: McGraw-Hill, Inc., 1974.

The killing efficiency can be adjusted by changing the flow rate. Increasing the flow rate will not change the flow streamline or affect the collision probability but will increase the impact force when the cells collide the nanospikes covered micropillar. A higher impact force can cause a more serious damage to the cells, as shown in the following figure from the paper presented by Carlo et. al. (2).

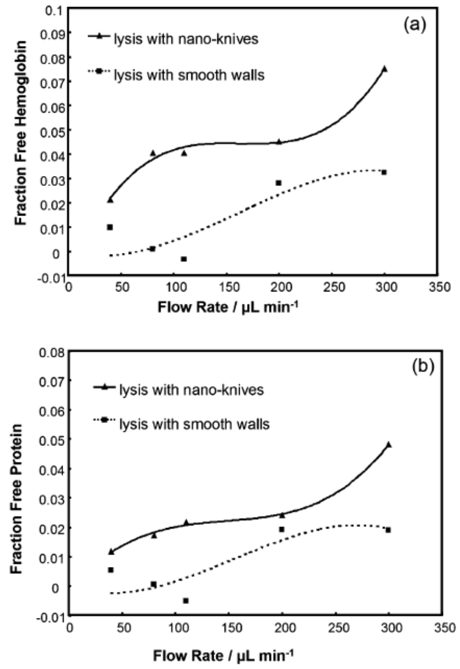

Fig. 5 The fraction of free hemoglobin (a) measured at 414 nm and free protein (b) measured at 280 nm absorbance are plotted as a function of volumetric flow rate for a single device. Results from a nano-knife modified device are compared to a smooth walled device. Curves are drawn to aid the eye. Negative values indicate less protein than in an unlysed centrifuged sample. Error of optical measurements is below 1%.

(2) *Carlo, D.D., Jeong, K., & Lee, L.P. (2003). Reagentless mechanical cell lysis by nanoscale barbs in microchannels for sample preparation. Lab on a chip, 3, 287-291.*
